# Polymorphic structures of 3-phenyl-1*H*-1,3-benzo­diazol-2(3*H*)-one

**DOI:** 10.1107/S2056989023003961

**Published:** 2023-05-12

**Authors:** Dabeen Hong, Kyounghoon Lee

**Affiliations:** a Department of Chemical Education and Research Institute of Natural Sciences, Gyeongsang National University, Gyeongsangnam-do 52828, Republic of Korea; Katholieke Universiteit Leuven, Belgium

**Keywords:** crystal structure, benzimidazolone, hydrogen bond

## Abstract

The polymorphic structures (**I** and **II**) of 3-phenyl-1*H*-1,3-benzo­diazol-2(3*H*)-one, C_13_H_10_N_2_O, acquired from pentane diffusion into the solution in THF, are reported. The structures show negligible differences in bond distances and angles, but the C—N—C—C torsion angles between the backbone and the phenyl substituent, 123.02 (15)° for **I** and 137.18 (11)° for **II**, are different. Compound **I** features a stronger C=O⋯H—N hydrogen bond than that in **II**, while the structure of **II** exhibits a stronger π–π inter­action than in **I**, as confirmed by the shorter inter­centroid distance [3.3257 (8) Å in **II** in comparison to 3.6862 (7) Å in **I**]. Overall, the supra­molecular inter­actions of **I** and **II** are distinct, presumably originating from the variation in the dihedral angle.

## Abstract 2

The two polymorphic structures of 3-phenyl-1*H*-1,3-benzo­diazol-2(3*H*)-one (**I** and **II**) exhibit identical bond distances and angles except for the C—N—C—C torsion angle between the benzimidazolone backbone and the phenyl substituent, which has an effect on the crystal packing and supra­molecular features. The structure of **I** contains a stronger C=O⋯H—N hydrogen-bonding inter­action and a weaker π–π inter­action between adjacent bezimidazolone moieties in comparison to **II**.

## 1. Chemical context

Benzimidazolo­nes are widely found in functional organic and biologically active mol­ecules (Palin *et al.*, 2008 ▸; Monforte *et al.*, 2010 ▸; Pribut *et al.*, 2019 ▸; Bellenie *et al.*, 2020 ▸). For example, substituted benzimidazolones have been used as pigments due to their high fastness and resistance to light and weathering (Metz & Morgenroth, 2009 ▸). In addition, the biological activities of benzimidazolone derivatives have been tested for anti­cancer, HIV, pain regulation, *etc*. (Henning *et al.*, 1987 ▸; Elsinga *et al.*, 1997 ▸; Tapia *et al.*, 1999 ▸; Kawamoto *et al.*, 2001 ▸; Poulain *et al.*, 2001 ▸; Roger *et al.*, 2003 ▸; Dombroski *et al.*, 2004 ▸; Gustin *et al.*, 2005 ▸; Li *et al.*, 2005 ▸; Hammach *et al.*, 2006 ▸; Monforte *et al.*, 2009 ▸).

Singly N-substituted benzimidazolo­nes exhibit inter­esting properties partially due to the hydrogen-bonding inter­actions between N—H⋯O=C moieties. *N*-phenyl-substituted benzimidazolone can be prepared by the intra­molecular *N*-aryl­ation of urea (Beyer *et al.*, 2011 ▸), carbonyl­ation of 2-nitro­aniline (Qi *et al.*, 2019 ▸), carbonyl­ation of *o*-phenyl­enedi­amine with CO_2_ (Yu *et al.*, 2013 ▸), carbonyl­ation of imino­phospho­rane with CO_2_ (Łukasik & Wróbel, 2016 ▸), iodo­syl­benzene-induced intra­molecular Hofmann rearrangement of 2-(phenyl­amino)­benzamide (Liu *et al.*, 2012 ▸), and carbonyl­ation of N1-phenyl­benzene-1,2-di­amine with 1,1′-carbonyl­diimidazole (Zhang *et al.*, 2008 ▸). Preparations of phenyl-substituted benzimidazolone have been reported using various reagents and catalysts, but the structure is unknown.

Here we report two polymorphic structures of 3-phenyl-1*H*-1,3-benzo­diazol-2(3*H*)-one. The compound was prepared following the reported procedure using 1,1′-carbonyl­diimidazole and N1-phenyl­benzene-1,2-di­amine in CH_2_Cl_2_ (Zhang *et al.*, 2008 ▸). Single crystals grown by pentane vapor diffusion into a THF solution formed colorless needles (**I**) and blocks (**II**). 
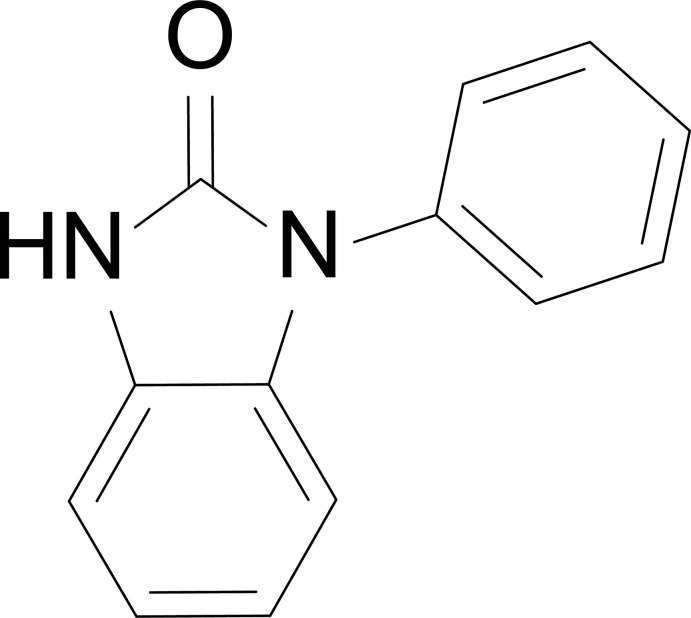


## 2. Structural commentary

**Figure 1:**
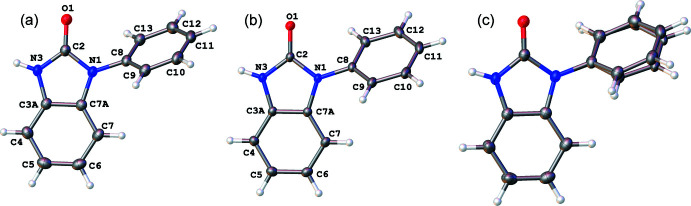
Mol­ecular structures of (*a*) **I**, (*b*) **II**, and (*c*) overlay of **I** and **II** with displacement ellipsoids drawn at the 50% probability level.

The title compounds crystallized as colorless needles (**I**) and blocks (**II**) in space groups *C*2/*c* and *Pbca*, respectively. The two polymorphic structures exhibit identical bond distances and angles, except for the dihedral angle of the phenyl substituent (Fig. 1 ▸). Both structures retain the planarity of benzimidazolone moiety, as demonstrated by the low r.m.s. deviations of 0.009 and 0.023 Å for **I** and **II**, respectively. The C2—N1—C8—C9/C13 torsion angle is 123.03 (14) and −137.18 (12)° for **I** and **II**, respectively. No additional differences are observed from an analysis of bond distances and angles.

## 3. Supra­molecular features

**Figure 2:**
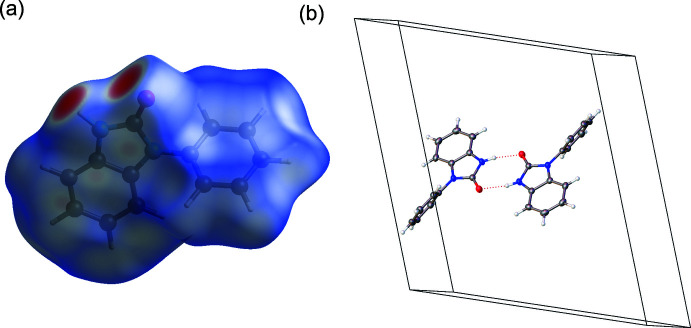
(*a*) Hirshfeld surface of **I** mapped over *d*_norm_. (*b*) Partial packing plot of **I**.

Initial investigations of supra­molecular features for **I** and **II** were carried out using Hirshfeld surface analysis with *CrystalExplorer 21.5* (Spackman *et al.*, 2021 ▸). The Hirshfeld surface was mapped over *d*_norm_ in the ranges −0.6415 to 1.2040 a.u. and −0.5612 to 1.1830 a.u. for **I** and **II**, respectively (Figs. 2 ▸ and 3 ▸). The most intense red spots on the surface for **I** and **II** indicate the N3—H3⋯O1 hydrogen-bonding inter­actions (Tables 1 ▸ and 2 ▸), which have 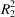 (8) graph-set motifs (Bernstein *et al.*, 1995 ▸). The shorter *D*⋯*A* and H⋯*A* distances, and more linear *D*—H⋯*A* angle reveal that the hydrogen-bonding inter­action in **I** is stronger than that in **II**. In contrast, the structure of **II** contains a stronger π–π inter­action between the adjacent benzimidazolone moieties, as defined by the centroid⋯centroid distance of 3.3257 (8) Å, while the corres­ponding distance in **I** is more elongated at 3.6862 (7) Å.

Minor inter­molecular inter­actions are observed as faint red spots on the surface. The spots in **I** indicate the inter­molecular inter­actions of C4⋯C2/C2⋯C4, C3*A*⋯C3*A* and C7—H7/H7—C7, whereas those in **II** correspond to C2⋯C5/C5⋯C2, C4—H4⋯C12/ C12⋯H4—C4, C7*A*⋯H6—C6/C6—H6⋯C7*A*, C3*A*⋯H6—C6/C6-H6⋯C3*A* and C3*A*⋯C6/C6⋯C3*A* contacts. The largest contributions to the Hirshfeld surface of **I** arises from H⋯H (44.4%), C⋯H/H⋯C (31.9%), and O⋯H/H⋯O (13.5%) contacts, whereas the contributions for **II** are H⋯H (45.8%), C⋯H/H⋯C (27.5%) and O⋯H/H⋯O (15.5%). Minor contributions include N⋯H/H⋯N (3.6%), C⋯C (3.2%), C⋯N/N⋯C (2.1%), C⋯O/O⋯C (1.4%) for **I** and C⋯C (5.4%), C⋯N/N⋯C (3.4%), N⋯H/H⋯N (3.2%), C⋯O/O⋯C (0.2%) for **II**.

## 4. Database survey

A search for the title compound in the Cambridge Structural Database (CSD, Version 5.43, update of November 2022; Groom *et al.*, 2016 ▸) did not match any reported structures, including aryl-derivative searches. However, a survey for mono-N-substituted benzimidazolone compounds revealed 75 results, which included structures with simple substituents such as methyl (WIKPAJ; Rong *et al.*, 2013 ▸), *tert*-butyl (WIKNOV; Rong *et al.*, 2013 ▸), octyl (ZANXET; Belaziz, Kandri Rodi, Essassi *et al.*, 2012 ▸), nonyl (IJUGIE; Ouzidan, Kandri Rodi *et al.*, 2011 ▸), decyl (ESANAQ; Ait Elmachkouri *et al.*, 2021 ▸), dodecyl (SECBUZ; Belaziz, Kandri Rodi, Ouazzani Chahdi *et al.*, 2012 ▸), benzyl (EVEYIO; Ouzidan, Essassi *et al.*, 2011 ▸), 4-methyl­benzyl (NEQBIW; Belaziz *et al.*, 2013 ▸), acetyl (VADYIM; Sebhaoui *et al.*, 2021 ▸) and a tri­fluoro­methyl group (ZEDJAX; Bouayad-Gervais *et al.*, 2022 ▸). Most structures feature bimolecular hydrogen-bonding inter­actions between N—H ⋯ O=C moieties with an 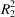 (8) graph-set motif, but in ZEDJAX N—H ⋯ O=C hydrogen bonds link the mol­ecules into *C*(4) chains. The distances between a nitro­gen donor and an oxygen acceptor range from 2.79–2.84 Å, comparable to the values for **I** and **II** of 2.7786 (14) and 2.8453 (14) Å, respectively.

## 5. Synthesis and crystallization

**Figure 4:**
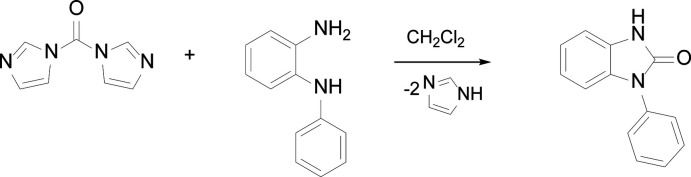
Synthesis of 3-phenyl-1*H*-1,3-benzo­diazol-2(3*H*)-one.

3-Phenyl-1*H*-1,3-benzo­diazol-2(3*H*)-one was prepared following a reported procedure (Fig. 4 ▸; Zhang *et al.*, 2008 ▸; Mark *et al.*, 2013 ▸). A solution of 1,1′-carbonyl­diimidazole (0.50 g, 3.1 mmol) and 2-amino­diphenyl­amine (0.57 g, 3.1 mmol) in CH_2_Cl_2_ (15 mL) was stirred at room temperature overnight. The resulting white precipitate was filtered. An additional white precipitate was acquired by adding Et_2_O (10 mL) into the filtrate. Combined yield: 0.30 g (46%). ^1^H NMR (CDCl_3_, 300 MHz): δ 10.75 (*br s*, NH, 1H), 7.58 (*m*, Ar, 4H), 7.45 (*m*, Ar, 1H), 7.17 (*m*, Ar, 1H), 7.10 (*m*, Ar, 1H), 7.06 (*m*, Ar, 2H). Pentane vapor diffusion into a solution of the compound in THF formed colorless needles and blocks.

## 6. Refinement

Crystal data, data collection, and refinement statistics are summarized in Table 3 ▸. No appreciable disorder was observed for both structures. The hydrogen atoms were optimized using riding models.

## Supplementary Material

Crystal structure: contains datablock(s) global, I, II. DOI: 10.1107/S2056989023003961/vm2281sup1.cif

Structure factors: contains datablock(s) I. DOI: 10.1107/S2056989023003961/vm2281Isup2.hkl

Structure factors: contains datablock(s) II. DOI: 10.1107/S2056989023003961/vm2281IIsup3.hkl

Supporting information file. DOI: 10.1107/S2056989023003961/vm2281Isup4.cml

CCDC references: 2260424, 2260423

Additional supporting information:  crystallographic information; 3D view; checkCIF report

## Figures and Tables

**Figure 3:**
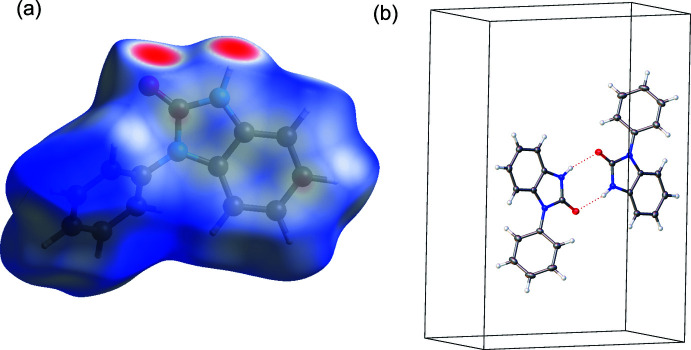
(*a*) Hirshfeld surface of **II** mapped over *d*_norm_. (*b*) Partial packing plot of **II**.

**Table 1:** Hydrogen-bond geometry (Å, °) for **I** [Chem scheme1]

*D*—H⋯*A*	*D*—H	H⋯*A*	*D*⋯*A*	*D*—H⋯*A*
N3—H3⋯O1^i^	0.88	1.91	2.7786 (14)	177

Symmetry code: (i) 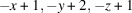 .

**Table 2:** Hydrogen-bond geometry (Å, °) for **II** [Chem scheme1]

*D*—H⋯*A*	*D*—H	H⋯*A*	*D*⋯*A*	*D*—H⋯*A*
N3—H3⋯O1^i^	0.88	2.00	2.8453 (13)	174

Symmetry code: (i) 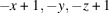 .

**Table 3:** Experimental details

	**I**	**II**
Crystal data		
Chemical formula	C_13_H_10_N_2_O	C_13_H_10_N_2_O
*M*_r_	210.23	210.23
Crystal system, space group	Monoclinic, *C*2/*c*	Orthorhombic, *P* *b* *c* *a*
Temperature (K)	193	193
*a*, *b*, *c* (Å)	18.0187 (9), 6.4455 (3), 18.7315 (10)	13.7925 (3), 7.2652 (1), 19.7956 (4)
α, β, γ (°)	90, 111.181 (3), 90	90, 90, 90
*V* (Å^3^)	2028.50 (18)	1983.62 (6)
*Z*	8	8
Radiation type	Mo *K*α	Mo *K*α
μ (mm^−1^)	0.09	0.09
Crystal size (mm)	0.51 × 0.23 × 0.14	0.37 × 0.33 × 0.19

Data collection		
Diffractometer	Bruker APEXII CCD	Bruker APEXII CCD
Absorption correction	Multi-scan (*SADABS*; Krause *et al.*, 2015 ▸)	Multi-scan (*SADABS*; Krause *et al.*, 2015 ▸)
*T*_min_, *T*_max_	0.699, 0.746	0.712, 0.746
No. of measured, independent and observed [*I* > 2σ(*I*)] reflections	9328, 2350, 1956	34068, 2479, 2203
*R*_int_	0.031	0.036
(sin θ/λ)_max_ (Å^−1^)	0.651	0.668

Refinement		
*R*[*F* ^2^ > 2σ(*F* ^2^)], *wR*(*F* ^2^), *S*	0.041, 0.104, 1.07	0.039, 0.099, 1.02
No. of reflections	2350	2479
No. of parameters	145	145
H-atom treatment	H-atom parameters constrained	H-atom parameters constrained
Δρ_max_, Δρ_min_ (e Å^−3^)	0.18, −0.23	0.25, −0.38

Computer programs: *APEX2* and *SAINT* (Bruker, 2012 ▸), *SHELXT* (Sheldrick, 2015*a* ▸), *SHELXL* (Sheldrick, 2015*b* ▸) and *OLEX2* (Dolomanov *et al.*, 2009 ▸).

## supplementary crystallographic information

### 3-Phenyl-1H-1,3-benzodiazol-2(3H)-one (I) Crystal data

**Table:**

C_13_H_10_N_2_O	*F*(000) = 880
*M**_r_* = 210.23	*D*_x_ = 1.377 Mg m^−^^3^
Monoclinic, *C*2/*c*	Mo *K*α radiation, λ = 0.71073 Å
*a* = 18.0187 (9) Å	Cell parameters from 2753 reflections
*b* = 6.4455 (3) Å	θ = 2.3–27.5°
*c* = 18.7315 (10) Å	µ = 0.09 mm^−^^1^
β = 111.181 (3)°	*T* = 193 K
*V* = 2028.50 (18) Å^3^	NEEDLE, colourless
*Z* = 8	0.51 × 0.23 × 0.14 mm

### 3-Phenyl-1H-1,3-benzodiazol-2(3H)-one (I) Data collection

**Table:**

Bruker APEXII CCD diffractometer	1956 reflections with *I* > 2σ(*I*)
φ and ω scans	*R*_int_ = 0.031
Absorption correction: multi-scan (SADABS; Krause *et al.*, 2015)	θ_max_ = 27.6°, θ_min_ = 2.3°
*T*_min_ = 0.699, *T*_max_ = 0.746	*h* = −23→23
9328 measured reflections	*k* = −8→8
2350 independent reflections	*l* = −24→24

### 3-Phenyl-1H-1,3-benzodiazol-2(3H)-one (I) Refinement

**Table:**

Refinement on *F*^2^	Primary atom site location: dual
Least-squares matrix: full	Hydrogen site location: inferred from neighbouring sites
*R*[*F*^2^ > 2σ(*F*^2^)] = 0.041	H-atom parameters constrained
*wR*(*F*^2^) = 0.104	*w* = 1/[σ^2^(*F*_o_^2^) + (0.0438*P*)^2^ + 1.4081*P*] where *P* = (*F*_o_^2^ + 2*F*_c_^2^)/3
*S* = 1.07	(Δ/σ)_max_ < 0.001
2350 reflections	Δρ_max_ = 0.18 e Å^−^^3^
145 parameters	Δρ_min_ = −0.23 e Å^−^^3^
0 restraints	

### 3-Phenyl-1H-1,3-benzodiazol-2(3H)-one (I) Special details

**Table:**

Geometry. All esds (except the esd in the dihedral angle between two l.s. planes) are estimated using the full covariance matrix. The cell esds are taken into account individually in the estimation of esds in distances, angles and torsion angles; correlations between esds in cell parameters are only used when they are defined by crystal symmetry. An approximate (isotropic) treatment of cell esds is used for estimating esds involving l.s. planes.

### 3-Phenyl-1H-1,3-benzodiazol-2(3H)-one (I) Fractional atomic coordinates and isotropic or equivalent isotropic displacement parameters (Å^2^)

**Table:**

	*x*	*y*	*z*	*U*_iso_*/*U*_eq_	
O1	0.60223 (5)	0.96614 (15)	0.57745 (5)	0.0239 (2)	
N3	0.53005 (6)	0.77512 (18)	0.46886 (6)	0.0210 (3)	
H3	0.488955	0.857863	0.448435	0.025*	
N1	0.64477 (6)	0.64976 (18)	0.54578 (6)	0.0199 (3)	
C8	0.71782 (7)	0.6314 (2)	0.60949 (7)	0.0200 (3)	
C7A	0.61292 (7)	0.5048 (2)	0.48703 (7)	0.0198 (3)	
C3A	0.54019 (7)	0.5865 (2)	0.43844 (7)	0.0202 (3)	
C2	0.59267 (7)	0.8152 (2)	0.53505 (7)	0.0201 (3)	
C9	0.73216 (8)	0.4585 (2)	0.65660 (7)	0.0242 (3)	
H9	0.694564	0.348538	0.645522	0.029*	
C13	0.77316 (7)	0.7904 (2)	0.62403 (7)	0.0230 (3)	
H13	0.763240	0.907155	0.590917	0.028*	
C7	0.64074 (8)	0.3170 (2)	0.47242 (8)	0.0236 (3)	
H7	0.689738	0.261391	0.506014	0.028*	
C4	0.49365 (8)	0.4809 (2)	0.37362 (7)	0.0238 (3)	
H4	0.443989	0.534926	0.340804	0.029*	
C5	0.52220 (8)	0.2924 (2)	0.35821 (8)	0.0274 (3)	
H5	0.491700	0.217059	0.313667	0.033*	
C10	0.80220 (8)	0.4481 (2)	0.72020 (8)	0.0275 (3)	
H10	0.812386	0.330940	0.753147	0.033*	
C6	0.59439 (8)	0.2116 (2)	0.40654 (8)	0.0266 (3)	
H6	0.612378	0.082452	0.394429	0.032*	
C12	0.84327 (8)	0.7775 (2)	0.68750 (8)	0.0274 (3)	
H12	0.881670	0.885313	0.697783	0.033*	
C11	0.85719 (8)	0.6075 (2)	0.73576 (7)	0.0283 (3)	
H11	0.904654	0.600345	0.779703	0.034*	

### 3-Phenyl-1H-1,3-benzodiazol-2(3H)-one (I) Atomic displacement parameters (Å^2^)

**Table:**

	*U*^11^	*U*^22^	*U*^33^	*U*^12^	*U*^13^	*U*^23^
O1	0.0232 (5)	0.0213 (5)	0.0244 (5)	0.0026 (4)	0.0052 (4)	−0.0008 (4)
N3	0.0185 (5)	0.0202 (6)	0.0217 (5)	0.0040 (4)	0.0041 (4)	0.0023 (4)
N1	0.0177 (5)	0.0193 (6)	0.0223 (5)	0.0027 (4)	0.0068 (4)	0.0014 (4)
C8	0.0177 (6)	0.0243 (8)	0.0193 (6)	0.0038 (5)	0.0083 (5)	0.0019 (5)
C7A	0.0191 (6)	0.0213 (7)	0.0216 (6)	−0.0015 (5)	0.0105 (5)	0.0018 (5)
C3A	0.0205 (6)	0.0207 (7)	0.0218 (6)	0.0006 (5)	0.0106 (5)	0.0032 (5)
C2	0.0195 (6)	0.0207 (7)	0.0209 (6)	0.0012 (5)	0.0081 (5)	0.0031 (5)
C9	0.0234 (6)	0.0248 (8)	0.0269 (7)	0.0031 (6)	0.0122 (5)	0.0037 (6)
C13	0.0221 (6)	0.0232 (8)	0.0244 (6)	0.0019 (5)	0.0095 (5)	0.0030 (5)
C7	0.0216 (6)	0.0236 (8)	0.0300 (7)	0.0022 (5)	0.0145 (5)	0.0029 (6)
C4	0.0229 (6)	0.0272 (8)	0.0210 (6)	−0.0022 (6)	0.0076 (5)	0.0025 (5)
C5	0.0333 (7)	0.0278 (9)	0.0251 (7)	−0.0077 (6)	0.0152 (6)	−0.0037 (6)
C10	0.0300 (7)	0.0295 (9)	0.0239 (6)	0.0100 (6)	0.0109 (5)	0.0071 (6)
C6	0.0322 (7)	0.0221 (8)	0.0327 (7)	−0.0013 (6)	0.0203 (6)	−0.0021 (6)
C12	0.0220 (6)	0.0306 (9)	0.0288 (7)	−0.0010 (6)	0.0083 (5)	−0.0044 (6)
C11	0.0232 (6)	0.0369 (9)	0.0211 (6)	0.0088 (6)	0.0037 (5)	−0.0022 (6)

### 3-Phenyl-1H-1,3-benzodiazol-2(3H)-one (I) Geometric parameters (Å, º)

**Table:**

O1—C2	1.2282 (16)	C13—H13	0.9500
N3—H3	0.8800	C13—C12	1.3896 (18)
N3—C3A	1.3824 (18)	C7—H7	0.9500
N3—C2	1.3660 (16)	C7—C6	1.3920 (19)
N1—C8	1.4266 (15)	C4—H4	0.9500
N1—C7A	1.3988 (17)	C4—C5	1.390 (2)
N1—C2	1.3864 (17)	C5—H5	0.9500
C8—C9	1.3868 (19)	C5—C6	1.390 (2)
C8—C13	1.3867 (19)	C10—H10	0.9500
C7A—C3A	1.4004 (17)	C10—C11	1.384 (2)
C7A—C7	1.375 (2)	C6—H6	0.9500
C3A—C4	1.3803 (18)	C12—H12	0.9500
C9—H9	0.9500	C12—C11	1.384 (2)
C9—C10	1.3893 (18)	C11—H11	0.9500
			
C3A—N3—H3	124.7	C12—C13—H13	120.3
C2—N3—H3	124.7	C7A—C7—H7	121.3
C2—N3—C3A	110.58 (11)	C7A—C7—C6	117.46 (12)
C7A—N1—C8	126.52 (11)	C6—C7—H7	121.3
C2—N1—C8	123.83 (11)	C3A—C4—H4	121.3
C2—N1—C7A	109.60 (10)	C3A—C4—C5	117.46 (12)
C9—C8—N1	120.15 (12)	C5—C4—H4	121.3
C13—C8—N1	118.93 (12)	C4—C5—H5	119.3
C13—C8—C9	120.91 (12)	C4—C5—C6	121.45 (13)
N1—C7A—C3A	106.37 (12)	C6—C5—H5	119.3
C7—C7A—N1	131.98 (12)	C9—C10—H10	119.9
C7—C7A—C3A	121.64 (12)	C11—C10—C9	120.28 (14)
N3—C3A—C7A	107.14 (11)	C11—C10—H10	119.9
C4—C3A—N3	131.89 (12)	C7—C6—H6	119.5
C4—C3A—C7A	120.97 (13)	C5—C6—C7	121.00 (14)
O1—C2—N3	127.84 (12)	C5—C6—H6	119.5
O1—C2—N1	125.88 (11)	C13—C12—H12	120.0
N3—C2—N1	106.28 (11)	C11—C12—C13	120.03 (13)
C8—C9—H9	120.4	C11—C12—H12	120.0
C8—C9—C10	119.16 (14)	C10—C11—C12	120.23 (12)
C10—C9—H9	120.4	C10—C11—H11	119.9
C8—C13—H13	120.3	C12—C11—H11	119.9
C8—C13—C12	119.38 (13)		
			
N3—C3A—C4—C5	178.80 (13)	C3A—N3—C2—O1	−178.55 (13)
N1—C8—C9—C10	−177.25 (12)	C3A—N3—C2—N1	1.45 (14)
N1—C8—C13—C12	177.74 (12)	C3A—C7A—C7—C6	0.98 (19)
N1—C7A—C3A—N3	−0.29 (13)	C3A—C4—C5—C6	0.7 (2)
N1—C7A—C3A—C4	179.34 (11)	C2—N3—C3A—C7A	−0.73 (14)
N1—C7A—C7—C6	−178.35 (12)	C2—N3—C3A—C4	179.69 (13)
C8—N1—C7A—C3A	178.81 (11)	C2—N1—C8—C9	123.03 (14)
C8—N1—C7A—C7	−1.8 (2)	C2—N1—C8—C13	−55.86 (17)
C8—N1—C2—O1	0.7 (2)	C2—N1—C7A—C3A	1.19 (14)
C8—N1—C2—N3	−179.32 (11)	C2—N1—C7A—C7	−179.40 (13)
C8—C9—C10—C11	−0.6 (2)	C9—C8—C13—C12	−1.1 (2)
C8—C13—C12—C11	−0.3 (2)	C9—C10—C11—C12	−0.8 (2)
C7A—N1—C8—C9	−54.27 (17)	C13—C8—C9—C10	1.62 (19)
C7A—N1—C8—C13	126.85 (14)	C13—C12—C11—C10	1.3 (2)
C7A—N1—C2—O1	178.38 (12)	C7—C7A—C3A—N3	−179.77 (11)
C7A—N1—C2—N3	−1.62 (14)	C7—C7A—C3A—C4	−0.14 (19)
C7A—C3A—C4—C5	−0.72 (19)	C4—C5—C6—C7	0.1 (2)
C7A—C7—C6—C5	−0.96 (19)		

### 3-Phenyl-1H-1,3-benzodiazol-2(3H)-one (I) Hydrogen-bond geometry (Å, º)

**Table:**

*D*—H···*A*	*D*—H	H···*A*	*D*···*A*	*D*—H···*A*
N3—H3···O1^i^	0.88	1.91	2.7786 (14)	177

Symmetry code: (i) −*x*+1, −*y*+2, −*z*+1.

### (II) Crystal data

**Table:**

C_13_H_10_N_2_O	*D*_x_ = 1.408 Mg m^−^^3^
*M**_r_* = 210.23	Mo *K*α radiation, λ = 0.71073 Å
Orthorhombic, *P**b**c**a*	Cell parameters from 9912 reflections
*a* = 13.7925 (3) Å	θ = 2.5–28.3°
*b* = 7.2652 (1) Å	µ = 0.09 mm^−^^1^
*c* = 19.7956 (4) Å	*T* = 193 K
*V* = 1983.62 (6) Å^3^	BLOCK, colourless
*Z* = 8	0.37 × 0.33 × 0.19 mm
*F*(000) = 880	

### (II) Data collection

**Table:**

Bruker APEXII CCD diffractometer	2203 reflections with *I* > 2σ(*I*)
φ and ω scans	*R*_int_ = 0.036
Absorption correction: multi-scan (SADABS; Krause *et al.*, 2015)	θ_max_ = 28.4°, θ_min_ = 2.1°
*T*_min_ = 0.712, *T*_max_ = 0.746	*h* = −18→18
34068 measured reflections	*k* = −8→9
2479 independent reflections	*l* = −26→26

### (II) Refinement

**Table:**

Refinement on *F*^2^	Primary atom site location: dual
Least-squares matrix: full	Hydrogen site location: inferred from neighbouring sites
*R*[*F*^2^ > 2σ(*F*^2^)] = 0.039	H-atom parameters constrained
*wR*(*F*^2^) = 0.099	*w* = 1/[σ^2^(*F*_o_^2^) + (0.0401*P*)^2^ + 1.3866*P*] where *P* = (*F*_o_^2^ + 2*F*_c_^2^)/3
*S* = 1.02	(Δ/σ)_max_ = 0.001
2479 reflections	Δρ_max_ = 0.25 e Å^−^^3^
145 parameters	Δρ_min_ = −0.37 e Å^−^^3^
0 restraints	

### (II) Special details

**Table:**

Geometry. All esds (except the esd in the dihedral angle between two l.s. planes) are estimated using the full covariance matrix. The cell esds are taken into account individually in the estimation of esds in distances, angles and torsion angles; correlations between esds in cell parameters are only used when they are defined by crystal symmetry. An approximate (isotropic) treatment of cell esds is used for estimating esds involving l.s. planes.

### (II) Fractional atomic coordinates and isotropic or equivalent isotropic displacement parameters (Å^2^)

**Table:**

	*x*	*y*	*z*	*U*_iso_*/*U*_eq_	
O1	0.45568 (7)	0.03382 (12)	0.59030 (4)	0.0202 (2)	
N1	0.38806 (7)	0.32974 (13)	0.58960 (5)	0.0156 (2)	
N3	0.43199 (7)	0.20514 (13)	0.49242 (5)	0.0162 (2)	
H3	0.452728	0.125653	0.462151	0.019*	
C7A	0.37019 (8)	0.46038 (16)	0.53914 (6)	0.0150 (2)	
C2	0.42885 (8)	0.17358 (16)	0.56054 (6)	0.0162 (2)	
C8	0.37484 (8)	0.35772 (16)	0.66031 (6)	0.0164 (2)	
C3A	0.39783 (8)	0.38031 (16)	0.47791 (6)	0.0152 (2)	
C7	0.33606 (8)	0.63906 (16)	0.54215 (6)	0.0176 (2)	
H7	0.319418	0.694844	0.583974	0.021*	
C4	0.38813 (8)	0.47304 (17)	0.41748 (6)	0.0178 (2)	
H4	0.405896	0.417587	0.375818	0.021*	
C5	0.35117 (8)	0.65146 (17)	0.42007 (6)	0.0195 (2)	
H5	0.342230	0.718091	0.379252	0.023*	
C9	0.44915 (9)	0.31474 (17)	0.70512 (6)	0.0198 (2)	
H9	0.507674	0.261337	0.689104	0.024*	
C13	0.28837 (9)	0.43311 (16)	0.68360 (6)	0.0203 (3)	
H13	0.237403	0.460514	0.652926	0.024*	
C6	0.32707 (8)	0.73401 (17)	0.48132 (6)	0.0192 (2)	
H6	0.304090	0.857272	0.481620	0.023*	
C12	0.27728 (10)	0.46798 (17)	0.75227 (7)	0.0257 (3)	
H12	0.218579	0.520089	0.768528	0.031*	
C11	0.35128 (11)	0.42719 (17)	0.79701 (6)	0.0268 (3)	
H11	0.343401	0.451607	0.843835	0.032*	
C10	0.43697 (10)	0.35064 (18)	0.77346 (6)	0.0245 (3)	
H10	0.487637	0.322592	0.804296	0.029*	

### (II) Atomic displacement parameters (Å^2^)

**Table:**

	*U*^11^	*U*^22^	*U*^33^	*U*^12^	*U*^13^	*U*^23^
O1	0.0276 (5)	0.0164 (4)	0.0168 (4)	0.0051 (3)	0.0006 (3)	−0.0002 (3)
N1	0.0173 (4)	0.0150 (4)	0.0145 (4)	0.0021 (4)	0.0005 (3)	−0.0022 (4)
N3	0.0188 (5)	0.0157 (5)	0.0142 (4)	0.0028 (4)	0.0004 (4)	−0.0022 (4)
C7A	0.0122 (5)	0.0172 (5)	0.0156 (5)	−0.0013 (4)	−0.0007 (4)	−0.0008 (4)
C2	0.0157 (5)	0.0168 (5)	0.0161 (5)	0.0004 (4)	0.0004 (4)	−0.0027 (4)
C8	0.0215 (5)	0.0137 (5)	0.0140 (5)	−0.0014 (4)	0.0023 (4)	−0.0020 (4)
C3A	0.0119 (5)	0.0160 (5)	0.0178 (5)	0.0001 (4)	−0.0001 (4)	−0.0025 (4)
C7	0.0148 (5)	0.0177 (5)	0.0203 (5)	0.0006 (4)	0.0012 (4)	−0.0035 (4)
C4	0.0166 (5)	0.0208 (6)	0.0161 (5)	0.0002 (4)	0.0004 (4)	−0.0011 (4)
C5	0.0167 (5)	0.0215 (6)	0.0202 (6)	−0.0001 (5)	−0.0006 (4)	0.0039 (5)
C9	0.0219 (6)	0.0199 (5)	0.0176 (5)	−0.0022 (5)	0.0005 (4)	0.0000 (4)
C13	0.0241 (6)	0.0162 (5)	0.0205 (6)	0.0009 (5)	0.0031 (5)	−0.0014 (4)
C6	0.0151 (5)	0.0166 (5)	0.0258 (6)	0.0011 (4)	0.0004 (4)	0.0006 (5)
C12	0.0353 (7)	0.0180 (6)	0.0237 (6)	0.0025 (5)	0.0117 (5)	−0.0024 (5)
C11	0.0460 (8)	0.0188 (6)	0.0156 (5)	−0.0047 (6)	0.0058 (5)	−0.0031 (5)
C10	0.0340 (7)	0.0230 (6)	0.0166 (6)	−0.0066 (5)	−0.0028 (5)	0.0013 (5)

### (II) Geometric parameters (Å, º)

**Table:**

O1—C2	1.2309 (14)	C4—H4	0.9500
N1—C7A	1.3997 (15)	C4—C5	1.3939 (17)
N1—C2	1.3908 (14)	C5—H5	0.9500
N1—C8	1.4262 (14)	C5—C6	1.3928 (17)
N3—H3	0.8800	C9—H9	0.9500
N3—C2	1.3685 (15)	C9—C10	1.3880 (17)
N3—C3A	1.3872 (15)	C13—H13	0.9500
C7A—C3A	1.3976 (15)	C13—C12	1.3912 (17)
C7A—C7	1.3821 (16)	C6—H6	0.9500
C8—C9	1.3910 (17)	C12—H12	0.9500
C8—C13	1.3911 (16)	C12—C11	1.383 (2)
C3A—C4	1.3793 (16)	C11—H11	0.9500
C7—H7	0.9500	C11—C10	1.387 (2)
C7—C6	1.3933 (17)	C10—H10	0.9500
			
C7A—N1—C8	125.54 (10)	C5—C4—H4	121.4
C2—N1—C7A	109.23 (9)	C4—C5—H5	119.3
C2—N1—C8	125.02 (10)	C6—C5—C4	121.32 (11)
C2—N3—H3	124.8	C6—C5—H5	119.3
C2—N3—C3A	110.31 (9)	C8—C9—H9	120.3
C3A—N3—H3	124.8	C10—C9—C8	119.35 (12)
C3A—C7A—N1	106.78 (10)	C10—C9—H9	120.3
C7—C7A—N1	131.77 (11)	C8—C13—H13	120.3
C7—C7A—C3A	121.41 (11)	C8—C13—C12	119.33 (12)
O1—C2—N1	126.63 (11)	C12—C13—H13	120.3
O1—C2—N3	126.89 (11)	C7—C6—H6	119.4
N3—C2—N1	106.48 (10)	C5—C6—C7	121.19 (11)
C9—C8—N1	119.98 (10)	C5—C6—H6	119.4
C9—C8—C13	120.59 (11)	C13—C12—H12	119.8
C13—C8—N1	119.41 (10)	C11—C12—C13	120.36 (12)
N3—C3A—C7A	107.15 (10)	C11—C12—H12	119.8
C4—C3A—N3	131.35 (11)	C12—C11—H11	120.0
C4—C3A—C7A	121.50 (11)	C12—C11—C10	119.96 (12)
C7A—C7—H7	121.4	C10—C11—H11	120.0
C7A—C7—C6	117.27 (11)	C9—C10—H10	119.8
C6—C7—H7	121.4	C11—C10—C9	120.41 (12)
C3A—C4—H4	121.4	C11—C10—H10	119.8
C3A—C4—C5	117.24 (11)		
			
N1—C7A—C3A—N3	−0.32 (12)	C8—N1—C7A—C3A	176.62 (10)
N1—C7A—C3A—C4	179.08 (10)	C8—N1—C7A—C7	−1.01 (19)
N1—C7A—C7—C6	179.56 (11)	C8—N1—C2—O1	3.36 (19)
N1—C8—C9—C10	177.03 (11)	C8—N1—C2—N3	−177.43 (10)
N1—C8—C13—C12	−177.13 (11)	C8—C9—C10—C11	0.53 (19)
N3—C3A—C4—C5	−179.65 (11)	C8—C13—C12—C11	−0.35 (19)
C7A—N1—C2—O1	178.21 (11)	C3A—N3—C2—O1	−178.39 (11)
C7A—N1—C2—N3	−2.59 (12)	C3A—N3—C2—N1	2.40 (12)
C7A—N1—C8—C9	−129.31 (12)	C3A—C7A—C7—C6	2.22 (16)
C7A—N1—C8—C13	48.81 (16)	C3A—C4—C5—C6	1.38 (17)
C7A—C3A—C4—C5	1.11 (17)	C7—C7A—C3A—N3	177.61 (10)
C7A—C7—C6—C5	0.27 (17)	C7—C7A—C3A—C4	−2.99 (17)
C2—N1—C7A—C3A	1.81 (12)	C4—C5—C6—C7	−2.11 (18)
C2—N1—C7A—C7	−175.82 (12)	C9—C8—C13—C12	0.98 (18)
C2—N1—C8—C9	44.70 (17)	C13—C8—C9—C10	−1.07 (18)
C2—N1—C8—C13	−137.18 (12)	C13—C12—C11—C10	−0.2 (2)
C2—N3—C3A—C7A	−1.31 (13)	C12—C11—C10—C9	0.1 (2)
C2—N3—C3A—C4	179.37 (12)		

### (II) Hydrogen-bond geometry (Å, º)

**Table:**

*D*—H···*A*	*D*—H	H···*A*	*D*···*A*	*D*—H···*A*
N3—H3···O1^i^	0.88	2.00	2.8453 (13)	174

Symmetry code: (i) −*x*+1, −*y*, −*z*+1.
